# Microsurgical Techniques Used to Construct the Vascularized and Neurotized Tissue Engineered Bone

**DOI:** 10.1155/2014/281872

**Published:** 2014-05-13

**Authors:** Junjun Fan, Long Bi, Dan Jin, Kuanhai Wei, Bin Chen, Zhiyong Zhang, Guoxian Pei

**Affiliations:** ^1^Department of Orthopedics Surgery, Xijing Hospital, Fourth Military Medical University, Xi'an 710032, China; ^2^Department of Orthopaedic Surgery, Southern Hospital, Southern Medical University, Guangzhou 510515, China; ^3^Department of Plastic and Reconstructive Surgery, Shanghai Ninth People's Hospital, Shanghai Key Laboratory of Tissue Engineering, School of Medicine, Shanghai Jiao Tong University, Shanghai 200011, China

## Abstract

The lack of vascularization in the tissue engineered bone results in poor survival and ossification. Tissue engineered bone can be wrapped in the soft tissue flaps which are rich in blood supply to complete the vascularization in vivo by microsurgical technique, and the surface of the bone graft can be invaded with new vascular network. The intrinsic vascularization can be induced via a blood vessel or an arteriovenous loop located centrally in the bone graft by microsurgical technique. The peripheral nerve especially peptidergic nerve has effect on the bone regeneration. The peptidergic nerve can be used to construct the neurotized tissue engineered bone by implanting the nerve fiber into the center of bone graft. Thus, constructing a highly vascularized and neurotized tissue engineered bone according with the theory of biomimetics has become a useful method for repairing the large bone defect. Many researchers have used the microsurgical techniques to enhance the vascularization and neurotization of tissue engineered bone and to get a better osteogenesis effect. This review aims to summarize the microsurgical techniques mostly used to construct the vascularized and neurotized tissue engineered bone.

## 1. Introduction

It is a big challenge for surgeons to repair the large bone defect caused by the trauma, infection, tumor, or other reasons. Autologous bone grafting is considered as the golden standard for the clinical treatment of bone defects [1–5], but there are many problems such as new wound to the donor site, limited source of autologous bone, and high risk of infection. In recent few decades, bone tissue engineering has developed very fast which gives us the hope to solve this problem. But there are still many problems with the clinical application of tissue engineered bone. Bone tissue is a highly vascularized tissue and the bone cells need sufficient blood supply to maintain their nutrition [6]. Before the vascularization completed, the implanted bone graft gets nutrition mainly through the osmosis of interstitium fluid and only 100–300 *μ*m thickness under the bone graft surface can get nutrition. The seed cells such as bone marrow stem cells in the center of the bone graft will lack enough nutrition and finally die. The osteogenesis is influenced by the process of vascularization and this limits the clinical application of tissue engineered bone. Over the last few years, many researchers have used the microsurgical techniques to enhance the vascularization of tissue engineered bone and get a better reparative effect in bone defect [7–10]. Tissue engineered bone grafts can be wrapped in the soft tissue flaps such as pedicle fascia flap, muscle flap, or periosteum flap to complete the vascularization in vivo by microsurgical technique and the surface of bone graft can be invaded with new vascular networks. The intrinsic vascularization can be induced via a blood vessel or an arteriovenous loop being implanted centrally in the bone graft by microsurgical technique. Our group also has successfully enhanced the vascularization of tissue engineered bone using these microsurgery techniques in many kinds of animal models from little animals such as rabbit to large animals such as goat and rhesus [11–13]. Besides the vascular networks, bone tissues also have abundant neural network distributed in the areas such as periosteum, marrow cavity, and bone cortex. The neural fibers also contact with the bone cells and medullary cells [14]. The peripheral nerve especially peptidergic nerve has the ability to regulate the function of osteoblasts and blood vessels [15–18]. So constructing the neurotized tissue engineered bone is very important to improve the reparative effect in bone defect. Our research group has successfully used the microsurgical techniques to achieve the neurotization of tissue engineered bone in vivo and improved the reparative effect on the bone defect [19–21]. We also have given the concept of constructing the highly vascularized and neurotized tissue engineered bone simultaneously by microsurgical techniques for repairing large bone defects according with the theory of biomimetics. This review aims to summarize the applications of microsurgical techniques mostly used to construct the vascularized and neurotized tissue engineered bone and to introduce our relative researches about this section.

## 2. Microsurgical Techniques Used for Vascularization

### 2.1. Tissue Flaps

Tissue flaps such as fascia flap, muscle flap, and peritoneum flap have rich blood supply and can be used as the vascular bed to enhance the vascularization of tissue engineered bone [22–24]. Before implanting the tissue engineered bone to the bone defect, we can wrap the bone graft in suitable tissue flap which is rich in blood supply to achieve the ectopic vascularization and osteogenesis at first and then implant the bone graft to the bone defect site with a reoperation [25–29]. Although this technique is feasible for the vascularization of tissue engineered bone, the pattern of new vascular network within the implanted bone graft is random, and the reoperation of bone graft to the bone defect site is impossible without damaging the initial vascular network [30]. To avoid the reoperation and shorten the treatment time, the vascular pedicled flaps such as pedicled fascia flap, pedicled muscle flap, and pedicled periosteum flap are used to achieve the vascularization of tissue engineered bone graft in vivo. These vascular pedicled flaps can be transferred to the bone defect site by microsurgical technique and wrap the tissue engineered bone in situ to achieve the vascularization without reoperation. Compared with the first method of ectopic vascularization, the method of vascularization in situ can shorten the treatment time and need no reoperation but is limited by the local circumstance of the bone defect site. Both methods need excellent microsurgical techniques.

There are several kinds of soft tissue flap that can be used to enhance the vascularization of tissue engineered bone. Fascia flap, muscle flap, and periosteum flap are the most commonly used to wrap the bone graft.

Fascia flap has rich capillary network and excellent permeability as a most suitable soft tissue flap for enhancing the vascularization. Our group is the first to report the use of fascia flap to enhance the vascularization of tissue engineered bone in the large animal model of goat. We found that pedicled fascia flap could accelerate the revascularization and osteogenesis process of tissue engineered bone in large animal model [12]. Liu et al. observed the bone formation using beagle deep fascia pedicled flap wrapping the tissue engineered bone and they found that the new blood vessels of tissue engineered bone in the fascia flap group were superior to those in the control group, suggesting that pedicled fascia flap could promote the revascularization of tissue engineered bone in vivo [31]. Yang et al. reported that the tissue engineered bone wrapped by the pedicled fascial flap had a sound reparative effect on bone defect due to its dual role of constructing vascularization and inducing membrane guided tissue regeneration [32].

Muscle flap also has rich blood supply and is usually used to repair the soft tissue defect or infected wound in clinical patients. The latissimus dorsi muscle is the most commonly used to wrap the tissue engineered bone. Casabona implanted a porous hydroxyapatite ceramic scaffold with the human bone marrow stromal cells into the latissimus dorsi of athymic mice and found the presence of new spongious bone tissue [33]. Terheyden used the xenogenic bone mineral as the bone scaffold and implanted it into a pouch prepared in the latissimus dorsi muscle of minipig. They found a large number of newly formed bone tissues and vascular networks [34]. Warnke implanted a custom bone graft into the latissimus dorsi muscle of an adult male patient and successfully repaired the mandibular bone defect of the patient [35].

Periosteum flap also has rich blood supply and can be used to induce bone regeneration. There are many reports about using the periosteum or cultured periosteal cells to enhance the osteogenesis and vascularization of tissue engineered bone [36–45]. A vascularized periosteal flap contains osteoprogenitor cells, structural matrix, and many kinds of growth factors such as bone morphogenetic protein. Vögelin et al. constructed a vascularized bone graft by using a periosteal flap in a rat model and found significant new bone tissue and vascular network formed in the tissue engineered bone [46]. But the periosteal flap is hard to obtain and difficult to wrap large scale of bone graft.

Although tissue engineered bone grafts can be wrapped in the soft tissue flaps which are rich in blood supply to enhance the vascularization in vivo by microsurgical techniques, only the surface of bone graft can be invaded with new vascular networks and the pattern of the new blood vessels is random. The intrinsic vascularization of bone graft is still not achieved with this method of soft tissue flap wrapping.

### 2.2. Axial Blood Vessel

The intrinsic vascularization can be induced via a blood vessel located centrally in the bone graft by microsurgical technique. There are many reports about the use of the carotid artery, jugular vein, and saphenous vascular bundle in biomaterials resulting in splendid osteogenesis and vascularization of the bone grafts [47–50]. Surgeons can embed a suitable axial blood vessel into the center of bone graft to get a vascularized bone tissue and then transfer the vascularized bone graft to the bone defect site using microsurgical vascular anastomosis. Similar to free pedicled flaps, the implanted bone graft can be immediately vascularized. Cassell et al. reported that there were plenty of capillary collateral newborns to build the capillary network when the blood vessel was stimulated. This method has been used in the clinical cases to treat the patients with ischemic necrosis of femoral head and the wrist scaphoid nonunion, and it showed the positive results [51]. Pelissier et al. reported that embedding a vascular pedicle in the coralline scaffold could enhance the vascularization and osteogenesis after being implanted in ectopic intramuscular sites [52]. Our group also used this method of implanting blood vessel into the center of tissue engineered bone to enhance the vascularization and osteogenesis in many animal models from little animals such as rabbit to large animals such as rhesus [11, 13]. As we know, we are the first to report the use of blood vessel to enhance the vascularization of tissue engineered bone in a primate animal model of rhesus by microsurgical techniques and our research proves the feasibility of this method to be used in clinical patients.

### 2.3. Arteriovenous Loop

Although the method of embedding axial blood vessel is available for enhancing the intrinsic vascularization of tissue engineered bone, the clinical application may be limited by the length and anatomic location of blood vessel. So a new method of arteriovenous (AV) loop has been used to enhance the intrinsic vascularization. This AV loop has been proved to be superior compared to the axial blood vessel both in terms of neovascular density and capacity [53]. This method was firstly described by Erol and Spira, and they successfully constructed a prefabricated full-thickness skin graft in a rat model by using the arteriovenous vessel loop. Significant neovascular network was observed originating from the AV loop [54]. Cassell et al. implanted the arteriovenous loop into polycarbonate isolation chambers and they successfully constructed the axially vascularized tissue by inducing vascularization in polymer matrices [55]. Arkudas et al. reported that using an arteriovenous loop could promote the survival and differentiation of transplanted autologous osteoblasts [56]. The AV loop has minimal morbidity of donor site and can be created at any surgical site without regarding the vascular pedicle length. Ren hypothesized that a combination of cells, biomaterials, growth factors, and arteriovenous loop may be able to generate a large and vascularized tissue engineered bone in vivo eventually [57].

Like autologous bone grafts, the new vascularized bone graft can be transferred to the bone defect site using the microsurgical techniques. This method of embedding blood vessel or AV loop will facilitate the clinical application of tissue engineered bone and offer new therapeutic strategies for reconstruction of large bone defects.

## 3. Microsurgical Techniques Used for Neurotization

### 3.1. The Important Role of Neurotization

Bone tissue is not only a highly vascularized tissue but also a highly neurotized tissue. By the anatomy study of the bone tissue, many researches have proved that there were dense and intimate networks of nerve in the bone tissue and the nerve networks had contact with bone cells [58–62]. Our group also found that there were many nerve fibers in human bone tissue distributed in the periosteum, cortical bone, cancellous bone, and bone marrow [63]. Then many researchers found the intimate relationship between the bone formation and the nerve networks development and proved that nerve networks played a very important role in the process of bone formation [64–66]. The mechanism about how the nerve networks act on the bone tissue was studied by many researchers. Bjurholm observed the features of the nerve fibers in bone tissue and showed that they were mainly peptidergic nerve fibers and had a neuroendocrine influence on bone physiology [67]. Hill et al. further reported that the peptidergic nerve fibers mainly accompanied blood vessels, and a few nerve endings stretched out and stopped in other locations such as the periosteum cell layer or bone marrow cells including osteoblasts and osteoclasts and released neuropeptides to regulate the bone physiology [68–70]. The finding that both osteoblasts and osteoclasts expressed functional neuropeptide receptors further proved the important role of nerve network in bone regeneration [71]. These neuropeptides including calcitonin gene-related peptide (CGRP) and nerve peptide Y (NPY) enhance osteoblast proliferation in vitro and inhibit the bone resorbing activity of isolated osteoclasts by regulating different cytokines pathways [72–79]. These neuropeptides are also associated with the dilation and the constrictor of blood vessels through interaction with neuropeptide receptors that are present on the wall of blood vessel via endothelium-dependent or endothelium-independent mechanisms, depending on the blood vessel type [80–85]. Blood vessels in the bone are innervated by the autonomic nervous system and sensory nerve fibers to regulate the blood flow [86], and sensory neuropeptides participate in the regulation of angiogenesis [87]. So these neuropeptides have ability to regulate the function of osteoblasts and to control the blood flow, and this can explain why it is very important to construct the nerve networks in tissue engineered bone to improve the effect of bone formation.

### 3.2. Nerve Tracts

The ability of the peptidergic nerve fiber to regulate the function of osteoblasts and blood vessels can be used to construct the neurotized tissue engineered bone by embedding the nerve fiber into the center of bone graft [19–21]. As we know, our group is the first in the world to successfully construct the neurotized tissue engineered bone by microsurgery techniques and repair the large bone defect in animal model. We implanted the sensory nerve tract or motor nerve tract into the side groove of *β*-TCP scaffold to repair a 1.5 cm femur defect in the rabbit. The values of bone mineral density and the neuropeptide were significantly higher in sensory nerve group compared with the motor never group and the blank group. But the ossification and nervalization effects of motor nerve group showed no significant difference compared with that of blank group [21]. Our group also investigated the effect of saphenous nerve and sciatic nerve homogenates on the proliferation and calcification of rabbit osteoblasts in vitro. We found that saphenous nerve homogenates significantly promoted the proliferation, differentiation, ALP activity, and mineralization of rabbit osteoblasts, but sciatic nerve homogenates did not show any osteogenic effect on the cells [88]. These results showed that the osteogenesis and neurotization of tissue engineered bone were more closely related to sensory nerves. By implanting the tissue engineered bone with sensory nerve tracts or blood vessels to repair a 1.5 cm femur defect in the rabbit, we found that implanting sensory nerve tracts or blood vessels into tissue engineered bone, respectively, could both improve the osteogenesis and the early expression of neuropeptides [20]. Although the group with sensory nerve tracts showed a little weaker effect compared to the vascular group, embedding nerve tracts into the tissue engineered bone could still improve the neurotization and osteogenesis of the bone graft compared to those in blank group. And this result may relate to the neuropeptides originated from the implanted sensory nerve. In our study, the saphenous nerve tract was separated and cut from the distal end in order to make the distal end of the nerve free, and then it was implanted into the side groove of the *β*-TCP scaffold. This method could make the neuropeptide, which was produced by the neurons, release from the distal end more easily, and it acted on the BMSCs via its receptors to regulate the metabolism of the bone.

Our group also evaluated the different effects on the expression of calcitonin gene-related peptide (CGRP) and neuropeptide Y (NPY) between tissue engineered bone with blood vessel in vivo or with sensory nerve tract. We found implantation of blood vessel into tissue engineered bone could significantly improve the expressions of CGRP and NPY at the early 3 months compared with implantation of sensory tract into tissue-engineered bone, but the differences were not significant at 6 or 12 months postoperatively [19]. We also investigated whether the implantation of blood vessel and sensory nerve tract could affect the expressions of neurokinin 1 receptor (NK1R) and vasoactive intestinal peptide type 1 receptor (VIPR1). We found that both blood vessel and sensory nerve could significantly improve the expression levels of NK1R and VIPR1 compared with the blank group [89]. Our researches showed that both sensory nerve tracts and blood vessel could be used to construct the neurotized tissue engineered bone.

Although sensory nerves can enhance the osteogenesis of tissue engineered bone, the neurocyte also needs the oxygen and nutrients. The neurotization of tissue engineered bone needs the vascularization as the preconditions. Masaki connected a silicone gel tube to the sciatic nerves of mice and then placed a small artery that was adjacent to the sciatic nerve into the silicone gel tube through a longitudinal incision to provide oxygen and nutrients to the regenerated nerve [90]. The improved microenvironment in the silicone tube produced good regeneration results. Palmer inferred that neurogenesis generally occurred near blood vessels because of the satisfactory vascular microenvironment with sufficient blood supply [91]. Meanwhile, blood vessels in the bone are also innervated by the autonomic nervous system and sensory nerve fibers to regulate blood flow, and sensory neuropeptides participate in the regulation of angiogenesis [86, 87]. So there is highly intimate relationship between the blood vessel and nerve networks in the bone tissue. When constructing tissue engineered bone, it is important to ensure adequate blood supply and perfect innervation to promote survival of the bone graft. As we know, our group is the first to give the concept of constructing the highly vascularized and neurotized tissue engineered bone simultaneously according with the theory of biomimetics by microsurgery techniques. It may be a dramatic method to construct the vascularized and neurotized tissue engineered bone simultaneously by implanting both blood vessel and the sensor nerve tract into the center of bone graft with microsurgery techniques.

## 4. Conclusion

The lack of vascularization and neurotization within the tissue engineered bone results in poor ossification and limits the clinical application of tissue engineered bone. The vascularization of bone grafts can be promoted by soft tissue flap wrapping, blood vessel embedding, and arteriovenous loop embedding centrally in vivo by microsurgical techniques, but this method has the disadvantage of uncertain neurotization effect. The neurotization of bone grafts can be promoted by peptidergic nerve tract embedding centrally in vivo by microsurgical techniques, but this method also has the disadvantage of uncertain vascularization effect. So each method has the superiority and disadvantage. Combination of different methods will be necessary and useful to improve the reparative effect of tissue engineered bone in the large bone defect. We give the concept of constructing the highly vascularized and neurotized tissue engineered bone simultaneously by microsurgical techniques according with the theory of biomimetics. The microsurgical techniques will play a more and more important role in the development of bone tissue engineering and eventually achieve the wide clinical application of tissue engineered bone to repair the bone defect of patients.
